# Estimation of HPV prevalence in young women in Scotland; monitoring of future vaccine impact

**DOI:** 10.1186/1471-2334-13-519

**Published:** 2013-11-05

**Authors:** Kimberley Kavanagh, Katy Sinka, Kate Cuschieri, John Love, Alison Potts, Kevin GJ Pollock, Heather Cubie, Martin Donaghy, Chris Robertson

**Affiliations:** 1 Department of Mathematics and Statistics, University of Strathclyde, Glasgow G1 1XH, UK; 2 Health Protection Scotland, Glasgow G2 6QE, UK; 3 Department of Laboratory Medicine, Scottish HPV Reference Lab, Royal Infirmary of Edinburgh, NHS Lothian EH16 4SA, UK; 4 International Prevention Research Institute (iPRI), 69006, Lyon, France; 5 Human Papillomavirus Group and Scottish HPV Investigators Network (SHINe), MRC Centre for Reproductive Health, University of Edinburgh, Edinburgh EH16 4TJ, UK

**Keywords:** Human papilloma virus, Vaccination, Prevalence, Multiple infections

## Abstract

**Background:**

Estimation of pre-immunisation prevalence of HPV and distribution of HPV types is fundamental to understanding the subsequent impact of HPV vaccination. We describe the type specific prevalence of HPV in females aged 20–21 in Scotland who attended or defaulted from cervical screening using three specimen types; from attenders liquid based cytology and from defaulters urine or self-taken swabs.

**Methods:**

Residual liquid based cytology samples (*n* = 2148), collected from women aged 20–21 attending for their first smear were genotyped for HPV. A sample (*n* = 709) from women who had defaulted from screening was also made available for HPV testing through the use of postal testing kits (either urine samples (*n* = 378) or self-taken swabs (*n* = 331)). Estimates of prevalence weighted by deprivation, and for the postal testing kit, also by reminder status and specimen type were calculated for each HPV type. The distribution of HPV types were compared between specimen types and the occurrence of multiple high-risk infections examined. The influence of demographic factors on high-risk HPV positivity and multiple infections was examined via logistic regression.

**Results:**

The prevalence of any HPV in young women aged 20–21 was 32.2% for urine, 39.5% for self-taken swab, and 49.4% for LBC specimens. Infection with vaccine specific types (HPV 16, 18) or those associated with cross-protection (HPV 31, 33, 45, 51) was common. Individuals were more likely to test positive for high-risk HPV if they resided in an area of high deprivation or in a rural area. The overall distribution of HPV types did not vary between defaulters and attenders. Multiple infections occurred in 48.1% of high-risk HPV positive individuals. Excluding vaccine types the most common pairing was HPV 56 and 66.

**Conclusions:**

Understanding of the pre-immunisation prevalence of HPV in young women puts Scotland in a prime position to assess the early effect of vaccination as the first highly vaccinated cohorts of individuals enter the screening programme. Differences in results with different specimen types must be taken into account when monitoring the impact of vaccination programmes.

## Background

The prevalence and circulating genotypes of human papillomavirus (HPV) infection in different study populations has recently been reported [1-3]. The incentive for such studies is a need for better understanding of HPV infection, but additionally to provide information to enable the impact of key changes in prevention, detection and management of HPV-related disease. Of particular importance is the introduction of HPV vaccine and increasing availability and use of HPV tests within cervical screening programmes.

A better understanding of the natural history and epidemiology of HPV feeds into models of vaccine impact and cost effectiveness to inform decision-making on HPV immunisation and future changes to cervical screening. Defining a baseline against which to monitor the impact of vaccination provides a means to measure the earliest expected change - which is a decrease in the prevalence of vaccine-specific HPV.

The range of study populations, severity and types of associated pathology in target age groups means that few of the studies on HPV prevalence are generalizable. Estimates of HPV prevalence in the general population often come from studies that consider samples from cervical screening programmes due to ease of sampling and availability of a link to cytology/disease status [1,3]. These frequently include women across a wide age range. Such studies may however introduce a bias if the prevalence of HPV in those who default from screening differs substantially from those who attend. In Scotland, attendance at cervical screening is high overall (71.2% in the previous 3.5 years in 2012–13) but is lower in those aged 20–24 (53.5%) [4].

For an estimate of Cervarix® vaccine impact it is important to ascertain the effect of the vaccination programme on the whole population, with particular focus on the age group where these changes will be observed first. In Scotland, cervical screening currently begins at age 20, therefore it is one of the few countries in the world able to detect early impacts of the vaccine which was introduced in September 2008. The vaccine was implemented as routine in-school vaccination of those aged 12–13 with a catch-up campaign for all those aged 13–17 years old at the time [5] and achieved sustained high uptake [6]. In order to monitor the immunisation programme, a complementary, systematic national public health HPV surveillance programme was implemented in Scotland.

This paper describes the methods used to establish representative population-based estimates of HPV prevalence in unvaccinated women aged 20–21 in Scotland. By using two sampling methods, we examined the prevalence of HPV in those who attended for cervical screening and in those who did not and the distribution of HPV types detected. We aimed to improve the representativeness of the baseline HPV prevalence estimates and compare patterns between those who default from and those who attend for screening. In addition, we examined the effect of different specimen types upon estimates of HPV prevalence. The occurrence of multiple HPV types is examined with particular emphasis on co-incidence of high-risk vaccine types (16 and 18) with other high-risk types. Such estimates are fundamental to understanding the impact of the HPV vaccination in the general population, while validating the public health approach taken in Scotland.

## Methods

### Sampling and surveys

Scotland has a population of approximately 5.3 million [7] and an organised cytology based cervical screening programme. Screening age is currently 20 to 60 years and women are first invited to attend after their 20th birthday. Following this invitation, if they do not attend they are sent two reminder invitations at three monthly intervals and are classified as “defaulters” if they have not attended by three months following the second reminder. All women in Scotland eligible for cervical screening are recorded in the Scottish Cervical Screening Call and Recall System (SCCRS), a population-based information technology system, which supports the programme and contains pathology results, recall and management information.

### Population attending for screening

In 2009, anonymised residual liquid based cytology (LBC) samples from young women aged 20–21 years attending their first screening appointment were collected from all (11) NHS cytopathology laboratories in Scotland, which have national coverage. To achieve the desired sample size of 2,000 specimens, each laboratory collected over a two month period, staggered throughout the year, and selected samples from women born after 1st January 1988, with the number of samples collected proportionate to the population which the laboratory served.

### Population defaulting from screening

Self-taken specimens were obtained using a postal testing kit (PTK) for women who defaulted from attending their first screening appointment in 2008. From an anonymised data file of 15367 women, 5500 were randomly selected across all Health Board administration areas ensuring a geographically representative sample. Half of the randomly selected women were sent a urine test kit and half a self-taken vaginal swab kit. Details of the methodology are reported elsewhere [8].

### Data and linkage

All LBC, swab and urine specimens were labelled with a study identification number. The study ID was separately linked to SCCRS data using the unique Community Health Index identifier. Geographical datazone, derived from postcode of residence, was attributed to each record. HPV results were matched to each record and all personal identifiable information was then removed from the dataset prior to statistical analysis. The Scottish Index of Multiple deprivation (SIMD) quintiles (1: Most deprived; 5: Least deprived) and urban–rural six-fold classification were extracted from the Scottish Neighbourhood Statistics (http://www.sns.gov.uk) and linked to the dataset via datazone. The percentage of non-white residents in each datazone (“white” defined as White Scottish, White Irish, White Other British or Other White according to the ethnicity question in the 2001 census) was obtained from http://www.scrol.gov.uk/scrol/warehouse/NewWards_ER_OA.jsp for the census output areas and the aggregated up to data zones. As high concentrations of non-white individuals are clustered within a small number of datazones, this variable was categorised into quintiles.

As a large proportion of individuals reside in urban classes 1 (large urban) or 2 (other urban), reflecting the population distribution in Scotland, the urban–rural classification was recoded so that categories 3 (accessible small towns) and 4 (remote small towns) are combined to form a small town class and 5 (accessible rural) and 6 (remote rural) a rural class giving a suitable number of specimens in each level.

### HPV testing

All LBC samples were collected in ThinPrep media whereas swab samples were collected within M4RT media (Remel Products, Thermo Scientific, Lenexa, KS, USA). Urine was collected in 20 ml universals without any stabilisation or preservative buffer. A 5 ml aliquot of urine samples was centrifuged and the pellet washed twice with Phosphate Buffered Saline (PBS) before reconstitution in 1 ml of PBS prior to extraction. Self-taken swabs and residual LBC samples were vortexed and a 1 ml aliquot used for extraction. Automated extraction for both sample types was performed used the MDX media Kit (Qiagen Ltd, Manchester, UK). As this automated platform incorporates batch extraction in a 12 × 8 grid format 10% of all samples constituted HPV negative internal quality control material, the grid reference of which varied across batches as a contamination check. In addition previously tested positive control material was also included within each batch. HPV amplification and genotyping was performed using biotinylated GP5 + 6+ primers, prior to downstream genotyping using the Digene HPV Genotyping RH test (Qiagen). This assay is capable of detecting 18 high-risk or putatively high-risk types according to current IARC classification [9], specifically the 12 types in Group 1 with “carcinogenic” status: HPV 16,18,31,33,35,39,45,51,52,56,58,59, the single type in Group 2A with “probably carcinogenic” status: HPV 68 and 6 types from Group 2B with “possibly carcinogenic” status: HPV 26,53,66,73,82. Other HPV types, including types 6 and 11 are not differentiated but identified as “HPV other”.

Four grouped definitions were created; positive for any HPV type, positive for the vaccine types 16 and 18, positive for the HPV types where some vaccine cross-protection has been demonstrated – HPV 31, 33, 45 and 51 [10] and finally, positive for any high-risk type (Group 1 and Group 2A [9]). In addition, individuals with multiple infections (infected with more than one HPV type) were identified.

### Statistical analysis

The sample size was determined by the design of the long-term surveillance system, where the primary aim is to detect changes in HPV positivity over time. 2000 LBC samples were collected from unvaccinated women in the baseline year. The target sample size in the postal testing kit study was 1000 in order to give a 90% power to detect differences in HPV positivity of ± 6% points between screening defaulters and attenders. The actual sample achieved was 709, from a 13.2% response rate to the study, see [8] for more details.

To obtain representative estimates of HPV prevalence from the PTK sample, weights were calculated by raking which matches marginal distributions of a survey sample to known population margins [11]. Observations were weighted according to the distribution of SIMD, specimen type (urine or swab) and reminder status in the PTK sample. The LBC samples obtained were weighted proportionately to the distribution of SIMD in the cohort of young women aged 20–21 years available for sampling – the SCCRS extract.

The discrepancy in the distribution of HPV types detected by each of the specimen types was assessed using a simulation test using 1000 simulations as described in Cuschieri *et al.*[12]. The distribution of HPV types for each specimen type was compared to the overall distribution of HPV types from all specimens. The simulated p-value was calculated from the proportion of simulations with a discrepancy greater that that observed in the data. In such a way it can be established if different patterns in HPV type positivity are found between the specimen types and therefore between screening defaulters and attenders.

The odds of HPV infection given urban/rural classification, the proportion of non-white residents and SIMD of the datazone of residence were estimated in both a univariable and multivariable model using survey weighted logistic regression adjusting for the specimen type – LBC, self-taken swab or urine – and using a linear trend test for the ordered variables. Interactions between each of SIMD, the proportion of non-white residents and urban/rural classification with specimen type were examined.

All analysis was carried out using the statistical software, R version 2.12.2 [13] with use of the survey library [14].

#### Ethics

Self-taken sample collection was approved by West of Glasgow Ethics Committee [09/S0703/13]. National surveillance has been approved through the NHS National Clinical Governance committees and Caldicott Guardians at individual NHS Boards. Data linkage of information was approved by the NHS National Services Scotland (NSS) CHI advisory group.

## Results

### Demographics

Characteristics of the cervical screening and self-sampling groups are shown in Table 1. HPV test results were available for 2148 residual LBCs and 709 self-taken samples from defaulters consisting of 331 swab and 378 urine samples. SIMD could not be linked to 86 of the LBC samples which were subsequently excluded from the analysis.

**Table 1:** Comparison those attending their first screening appointment, defaulters who provided a self-taken sample, and the SCCRS cohort

	**Attenders**		**Defaulters**		**Cohort**	
	**Liquid based cytology samples**		**Self-taken samples**		**Extract of registered women from SCCRS**	
	**n**	**%**	**n**	**%**	**n**	**%**
*SIMD*						
1: Most deprived	470	22.8	145	20.5	9204	22.3
2	481	23.3	125	17.6	8689	21.1
3	400	19.4	129	18.2	7515	18.2
4	333	16.1	147	20.7	7262	17.6
5: Least deprived	378	18.3	163	23.0	8545	20.7
*Proportion of non-white residents in datazone*						
Q1 [0.0%-0.4%)	458	22.2	151	21.3		
Q2 [0.4%-1.0%)	450	21.8	137	19.3		
Q3 [1.0%-2.0%)	431	20.9	135	19.0		
Q4 [2.0%-5.3%)	398	19.3	136	19.2		
Q5 [5.3%-65.6%)	325	15.8	150	21.2		
*Datazone urban/rural*						
1: large urban	991	48.1	339	47.8		
2: other urban	626	30.4	196	27.6		
3-4: accessible/remote small towns	204	9.9	83	11.7		
5-6: accessible/remote rural	241	11.7	91	12.8		

SIMD is the Scottish Index of Multiple Deprivation. The proportion of non-white residents in each area is derived from 2001 census data. SCCRS is the Scottish Cervical Call/Recall System which all women eligible for screening in Scotland are recorded on.

### Weighted prevalence estimates and type distribution

The prevalence of any HPV was 32.2% for the urine, 39.5% for the self–taken swab, and 49.4% for the LBC samples (Table 2). Type 16 and/or 18 was detected in 10% of the urines, compared with 16.6% of the self-taken swabs and 23% of the LBCs. For any high-risk HPV, the respective prevalence estimates were 19.1%, 29.1% and 41.2%, for high-risk types excluding 16 and 18, 13.2%, 20.6% and 32.3% and for high-risk types excluding the cross protection types, 8%, 11.2% and 21.5%. The unadjusted odds of detection of high-risk HPV positivity was reduced for the self–taken swabs compared to the LBC samples (OR = 0.59 (95% CI: 0.45, 0.76), *p* = 0.001) and similarly for the urine samples (OR = 0.34 (95% CI: 0.26, 0.44), *p* < 0.0001) (Table 3).

**Table 2:** Weighted prevalence of HPV and corresponding 95% confidence interval (CI) stratified by sample type

	**Urine**		**Self-taken swab**		**LBC**	
**HPV type**	**Prevalence**	**95% CI**	**Prevalence**	**95% CI**	**Prevalence**	**95% CI**
Any HPV	32.2%	(27.4, 37.0)%	39.5%	(34.2, 44.9)%	49.4%	(47.2, 51.5)%
HPV 16 or 18	10.0%	(6.8, 13.1)%	16.6%	(12.5, 20.7)%	23.0%	(21.2, 24.8)%
HPV 31 or 33 or 45 or 51	9.3%	(6.4, 12.3)%	13.5%	(9.7, 17.3)%	19.1%	(17.4, 20.8)%
Any high-risk HPV	19.1%	(15.0, 23.1)%	29.1%	(24.1, 34.1)%	41.2%	(39.1, 43.4)%
High-risk excluding HPV 16 or 18	13.2%	(9.8, 16.7)%	20.6%	(16.2, 25.0)%	32.3%	(30.2, 34.3)%
High-risk excluding HPV 16, 18, 31, 33, 45 or 51	8.0%	(5.2, 10.8)%	11.2%	(7.7, 14.7)%	21.5%	(19.7, 23.2)%

PTK samples were weighted by SIMD, specimen type and reminder status. LBC samples were weighted by SIMD.

**Table 3:** Unadjusted and adjusted odds of infection with any high-risk HPV type for each specimen type, SIMD and urban/rural classification

	**Unadjusted OR**	**95% CI**	**Adjusted OR**	**95% CI**
***Specimen type***				
LBC	1	-	1	-
Self-taken swab	0.59	(0.45, 0.76)	0.58	(0..45, 0.75)
Urine	0.34	(0.26, 0.44)	0.33	(0.25,0.44)
***SIMD***				
1: Most deprived	1		1	
2	0.78	(0.62, 0.99)	0.73	(0.57, 0.93)
3	0.96	(0.75, 1.22)	0.87	(0.67, 1.12)
4	0.78	(0.60, 1.01)	0.69	(0.53, 0.91)
5 Least deprived	0.64	(0.49, 0.82)	0.62	(0.48, 0.80)
***Urban rural classification***				
1: Large urban	1	-	1	-
2: Other urban	1.38	(1.15, 1.67)	1.41	(1.16, 1.71)
3-4: accessible/remote small towns	1.19	(0.90, 1.57)	1.25	(0.94, 1.66)
5-6: accessible/remote rural	1.60	(1.24, 2.07)	1.68	(1.28, 2.20)
***Proportion of non-white residents in datazone***				
Q1 [0.0%-0.4%)	1	-		
Q2 [0.4%-1.0%)	0.89	(0.70, 1.14)		
Q3 [1.0%-2.0%)	0.84	(0.66, 1.08)		
Q4 [2.0%-5.3%)	0.72	(0.56, 0.92)		
Q5 [5.3%-65.6%)	0.56	(0.43, 0.73)		

**Figure 1:**
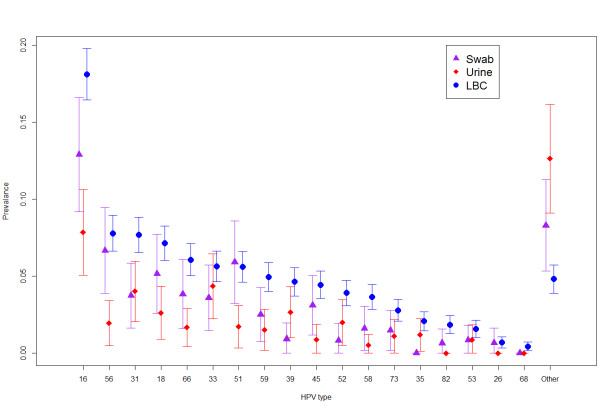
Weighted estimates of HPV prevalence stratified by sample type.

Type specific analyses (Figure 1) by specimen type demonstrate that type 16 is the most predominant HPV type with LBC samples having the highest prevalence (18.1% (95% CI: 16.4, 19.8%)). Generally the prevalence for each HPV type is highest for LBC samples, followed by self-taken swabs and then urine.

Comparison of the distributional spread of HPV types, excluding the “HPV other” category, showed that the aggregated PTK sample (urines and swabs together) did not have a significantly different distribution compared to the LBC samples (*p* = 0.470) indicating little difference in HPV types between those who defaulted and those who attended screening. Within the PTK sample, urine and self-taken swabs were found to have a different distribution of types (*p* = 0.032) with higher detection of HPV types 16, 56, 18, 51, and 45 in the self-taken swabs compared to urine.

### Which factors influence high-risk HPV positivity?

Adjusting for the effect of specimen type, urban/rural classification, SIMD and proportion of non-white residents were all found individually to have a statistically significant linear trend effect on positivity (p-value for urban/rural, *p* = 0.0029, proportion of non-white residents quintile, *p* < 0.0001, SIMD *p* = 0.002). No significant interactions were found (SIMD*specimen type, *p* = 0.119, proportion of non-white residents*specimen type, *p* = 0.08, urban/rural*specimen type, *p* = 0.858). In the multivariable model, the linear effect of quintile of non-white residents became statistically non-significant (*p* = 0.497) and was removed from the model.

The reduced model showed a linear effect of SIMD (*p* = 0.00071) and of urban/rural residence (*p* = 0.0014) and a difference between the specimen types (*p* < 0.0001). The adjusted odds ratios indicate that individuals in SIMD4 and SIMD5 (least deprived) had lower odds of high-risk HPV positivity than those in SIMD1 (most deprived) (Table 3). The odds of any high-risk HPV infection for individuals in rural areas (classes 5–6) were 1.68 times more than those in the large urban areas (class 1). There was an increase in the odds of high-risk HPV positivity for those living in other urban areas (class 2) in comparison to large urban areas (adjusted OR = 1.41(95% CI: 1.16, 1.66)). There is a strong association between deprivation and urban rural status. Of those individuals from large urban areas nearly 30% were highly deprived (SIMD1) compared to 22% overall while among those who live in remote rural areas there are few in SIMD 1 or 5. This association had no impact on the ORs of being high-risk HPV positive implying that the SIMD gradient associated with HPV positivity is the same in large urban areas as in other areas (interaction test *p* = 0.477).

### Multiple high-risk HPV infections

Overall, 1271 individuals tested positive for any HPV and 1021 for a high-risk HPV type. Of these 1021, 491 (48.1%) were infected with more than one high-risk type, 17.7% of the total samples (*n* = 2771). Of those infected with more than one high-risk HPV type, 58.4% were infected with two types, 25.6% with three, 9.9% with four, 4.3% with five and 1.8% with more than five. The maximum number found in an individual was 8 high-risk HPV types.

**Figure 2:**
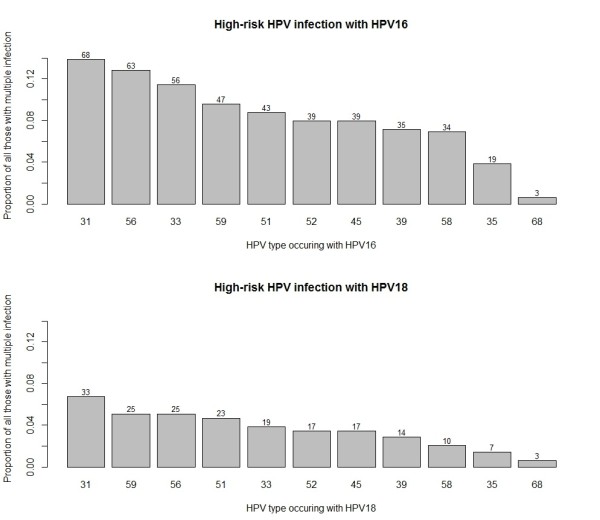
All high-risk HPV types occurring with HPV 16 (top panel, excluding HPV18) and HPV 18 (bottom panel, excluding HPV16) in those individuals with multiple high-risk HPV infections (n = 491).

HPV types most frequently found with the vaccine specific types are summarised in Figure 2. The vaccine specific pairing of types 16 and 18 occurred in 10.8% of those with multiple high-risk types. The most common pairings with type 16, excluding type 18, were high-risk HPV types 31, 56, 33 and 59. Types 31, 56 and 59 were also the most common with type 18. Limiting the analysis to the non-vaccine types, the most common pairing was types 56 and 66 (5.4% of those with multiple types).

Amongst those positive for any high-risk HPV no significant association was found between SIMD quintile (*p* = 0.76), urban/rural status (*p* = 0.46) or ethnicity quintile (*p* = 0.62) and the odds of having a multiple high-risk HPV infection compared to a single high-risk HPV. Differences were found in the odds of detecting multiple high-risk HPV infection between specimen types (*p* = 0.016) with fewer multiple infections detected in self-taken samples in line with the reduced sensitivity of these collection methods (Self taken swab OR = 0.61 (95% CI: 0.4, 0.96); Urine OR = 0.60 (95% CI:0.37, 0.98)).

## Discussion

It is a challenge to measure the prevalence of HPV since, unlike many other infections, there is comparatively little diagnostic type specific HPV testing which would allow surveillance through existing data sets such as public health notification systems. Many prevalence studies are restricted to populations that attend for cervical screening, those under investigation for cervical abnormalities detected or those accessing sexual health services [15,16]. In this study of unvaccinated 20 and 21 year olds in Scotland eligible for their first cervical screen, we have obtained samples from both those attending screening and defaulters, albeit the latter limited by a low response rate to the PTK -13.2% [8], in order to estimate pre-immunisation HPV prevalence in this cross-section of the population.

Our original aim was to compare HPV positivity among attenders for screening and defaulters but this comparison is confounded with specimen type. The difference in prevalence of 39.5% from the self-taken swab and 49.4% from the nurse-taken LBC swab suggests that those who do not attend for screening have a lower prevalence. This is unlikely to be the case. As this study shows a consistent gradient of increasing HPV positivity with increasing SIMD deprivation levels and the screening defaulters have a greater preponderance of SIMD1 (deprived) individuals compared to those who attend for screening [8] we would have anticipated greater HPV positivity among the defaulters. We therefore conclude that the differences are likely due to the different specimen types and the baseline HPV patterns within the defaulter population is likely to be similar to the attenders - consequently the on-going assessment of the impact of HPV vaccination in Scotland will be based on LBC samples.

HPV infection was common in this population of young women and multiple infections were detected in approximately half of all HPV positive samples. Baseline pre-immunisation levels of the vaccine specific types were high, with HPV 16 the more common than HPV 18. The aggregate occurrence of non-vaccine types, in particular those deemed to be high-risk, is more prevalent than the vaccine specific types and our results are comparable to those of other recent UK based studies [15,17].

Individual risk factors associated with HPV infection status, such as sexual activity and ethnic minority background [17], were not available. Linkage allowed assignment to each individual of SIMD, urban/rural designation and proportion of non-white residents. These should not be interpreted as individual attributes in this study. In a fully adjusted model, significant association with high-risk HPV positivity was found with increasing deprivation and rural classification. Although there has been some indication that individuals in rural areas may have higher levels of risky sexual behaviour [18,19] this is not well evidenced. However, this result is in accordance with the findings from the school based prevalence study carried out in Scotland which showed increasing HPV positivity with increasing levels of deprivation and in rural schools [20].

There was no overall difference in the distribution of HPV types between the aggregate PTK sample and the LBC samples, implying that HPV patterns are similar in both populations. In terms of prevalence estimates, urine and self-taken swabs are not comparable to LBC samples nor to each other but if used consistently could allow trends to be monitored for surveillance purposes especially in those defaulting from screening [21].

Multiple high-risk HPV infections were common, with around half of those infected having more than one type detected. Quantifying and defining the extent of multiple HPV infection is important as it may limit or enhance the effectiveness of the immunisation programme. With regard to the former, any increase in the prevalence of non-vaccine high-risk genotypes due to type replacement rather than unmasking could erode the programme’s cost-effectiveness. On the other hand, cross protection of the vaccine may somewhat mitigate against type replacement, particularly against 31, 33 & 45. There is some evidence suggesting that Cervarix® may confer relatively high levels of cross-protection [22].

Sexual behaviour has been shown to influence the risk of having multiple HPV infections and overall there are differences between younger and older women in factors that influence high-risk HPV positivity [23]. There is a slight age gap between the two populations of young women sampled (LBC and PTK). The LBC data were derived from young women aged 20 and 21 in 2009 (approximate 50:50 split between the ages) whilst the PTK were sent to women reaching 21 in 2008. There may have been a secular increase in overall HPV prevalence in the intervening time period. However, any effect that this may have had is likely to be minimal given that HPV infections can persist and given that we are measuring prevalence rather than incidence.

## Conclusions

Our study estimated the prevalence of HPV in unvaccinated young women living in Scotland. This study was essential in order to determine the baseline burden of HPV infection before vaccination. Specimens were tested using the same HPV assay at the same testing laboratory ensuring consistency of results. Although the varying sensitivity of the different specimen types in detecting HPV does not allow direct comparison between the prevalence of HPV in those attending screening and those defaulting, the similarity in the type specific HPV prevalence distributions and the consistent differences in the prevalence estimates obtained from the three sample types suggest that the infection pattern between defaulters and attenders is unlikely to be substantially different.

Due to the low uptake of defaulters to the PTK [8] the effectiveness of this tool for surveillance of changes in HPV prevalence post vaccination is limited and is therefore unlikely to be repeated. However, give the high and equitable uptake of HPV vaccination achieved in young women in Scotland [6], limiting surveillance to testing samples from those attending screening is likely to estimate accurately the effect of the vaccination on HPV prevalence in young women overall.

We are now in a prime position to ascertain the early effects of the HPV vaccine on young women who were part of the initial vaccination campaign and who were called for cervical screening from 2010. Having a systematic surveillance programme which has included the characterisation of a pre-vaccine baseline will allow us to robustly determine these effects.

## Competing interests

The authors declare that they have no competing interests.

## Authors’ contributions

KK conducted the statistical analysis and led the drafting of the paper with all authors. CR, KS, KC and HC designed the study. KC undertook HPV testing of the samples. JL managed and linked the data. KS, AP, KP and MD managed the programme and provided epidemiological interpretation. All authors read and approved the final manuscript.

## Pre-publication history

The pre-publication history for this paper can be accessed here:

http://www.biomedcentral.com/1471-2334/13/519/prepub
